# Ultra-Processed Foods: A Narrative Review of the Impact on the Human Gut Microbiome and Variations in Classification Methods

**DOI:** 10.3390/nu16111738

**Published:** 2024-06-01

**Authors:** Allison L. Brichacek, Melanie Florkowski, Esther Abiona, Karen M. Frank

**Affiliations:** Department of Laboratory Medicine, National Institutes of Health Clinical Center, Building 10, 10 Center Drive MSC 1508, Bethesda, MD 20892, USA; allison.brichacek@nih.gov (A.L.B.); melanie.florkowski@nih.gov (M.F.); esther.abiona@nih.gov (E.A.)

**Keywords:** ultra-processed food (UPF), gut, microbiota, microbiome, food classification, diet, clinical studies, NOVA

## Abstract

Ultra-processed foods (UPFs) are foods that are industrially processed and are often pre-packaged, convenient, energy-dense, and nutrient-poor. UPFs are widespread in the current Western diet and their proposed contribution to non-communicable diseases such as obesity and cardiovascular disease is supported by numerous studies. UPFs are hypothesized to affect the body in multiple ways, including by inducing changes in the gut microbiome. This review summarizes the available research on the effect of UPFs on the gut microbiome. We also review current usage of the NOVA food classification system in randomized controlled trials and observational studies and how its implementation effects UPF research. Despite some differences in methodology between studies, results often associate UPF consumption with a number of negative health consequences. There are attempts to standardize a UPF classification system; however, reaching and implementing a consensus is difficult. Future studies focusing on the mechanisms by which UPFs effect the body, including through the microbiome and metabolome, will be essential to refine our understanding of the effects of UPFs on human health.

## 1. Introduction

The Western diet is a modern dietary pattern in industrialized countries characterized as having high intake of processed and refined foods, red and processed meats, added sugars, and saturated and trans fats. Processed food types within this diet, such as fast food and ready-to-consume, pre-packaged foods are generally energy-dense, yet nutrient-poor, and account for a high percentage of daily caloric intake [1]. Pre-packaged processed foods are convenient in that they reduce the time required for cooking, are cost-effective, and are generally enjoyable to consume. The Western diet, coupled with a sedentary lifestyle, has been associated with chronic metabolic inflammation, which is thought to contribute to the development of many prevalent non-communicable diseases, including obesity, diabetes, cardiovascular disease (CVD), and cancer [2]. Studies of the health impact of ultra-processed foods (UPFs) overlap with studies of the impact of a Western diet, as a Western diet typically includes a high proportion of UPFs. There is growing evidence that UPFs are contributing to the increase in non-communicable diseases, morbidity, and mortality, through a number of potential mechanisms [3,4,5]. The mechanisms by which UPFs may cause harm include (1) increased total energy intake due to increased portion sizes and high caloric density, (2) increased glycemic response, (3) associated higher salt, sugar, and saturated fats that have been studied extensively, (4) additives that likely affect the gut microbiota and associated metabolism, (5) Maillard reaction products, acrolein and acrylamide, that have been associated with insulin resistance and oxidative stress, and (6) changes in food absorption due to altered food matrix and corresponding intestinal inflammation [6]. An additional mechanism to consider is the effect of UPFs and food additives on gut health through changes in the composition and metabolism of the gut microbiome [7]. There is accumulating evidence to suggest that a diet high in UPFs may disrupt the normal intestinal mucus barrier and goblet cell function [8]. Few studies have examined associations between UPFs and the gut microbiota; however, studies on the typical Western diet are able to offer some insight. This review summarizes the findings from studies associating the gut microbiota and (1) UPFs, (2) plant-based diets, (3) fast-food meals, and (4) ultra-processed meals/supplements with high nutritional value. Also included is a brief section on food additives, which are abundant in UPFs.

The most commonly used UPF classification system, NOVA, was first proposed in 2009 [9], was refined in 2016 [10], and has since been adopted by the Food and Agriculture Organization of the United Nations [11]. NOVA categorizes foods into one of four groups based on the extent and purpose of processing, rather than in terms of nutrients, such that group 1 items are non-processed or minimally processed items (e.g., fresh fruits and vegetables), group 2 are processed cooking ingredients (e.g., oil, flour, sugar), group 3 are processed items (e.g., some canned fruits, vegetables, or meats), and group 4 items are UPFs (e.g., packaged chips, cookies, pre-made meals, etc.) [10]. However, there has been much discussion on the challenges faced by researchers to apply this classification system in their clinical studies and debate on the ambiguous food items which potentially fit into more than one category. Furthermore, the methods used in dietary studies vary by collection tool (e.g., Food Frequency Questionnaire (FFQ), 24 h dietary recalls, food records) and frequency, which can add an additional layer of difficulty for comparing studies because the level of detail varies across the studies. Clinical nutritional studies with a focus on UPFs that have used NOVA have exponentially increased over the last five years, some of which have provided their interpretations and methods for discretionary analysis for classifying ‘difficult’ foods, and we summarize these approaches in this review.

The epidemiological and randomized controlled trials discussed in this review are vital to forming hypotheses for ongoing and future research; however, more studies are needed to warrant changes in public health policies. The central aims of this article are to (1) provide a review of the clinical evidence associating UPFs and gut microbiota alterations, and (2) to discuss a representative sampling of the individual detailed methods of researchers using the NOVA classification system in their clinical studies.

## 2. Methods

### 2.1. UPFs and the Gut Microbiome

For this narrative review, the articles included were chosen from searches in PubMed. The online searches were conducted using the following keywords: ultra-processed, processed, diet, food, Western, healthy, gut, microbiome, microbiota, fast food, meal, additive, and combinations thereof. Additional relevant publications were found in the citations of the articles found in our literature search. Publications were restricted to original human studies written in the English language. Selected articles contained both a method for classifying food processing level and reported sequencing data for the gut microbiome and can be viewed in Table 1.

### 2.2. UPF Classification Methods

Articles for discussion of UPF classification methods were found by searching the PubMed database. Searches were conducted using a set of search terms relating to food processing and ultra-processed foods. We excluded review articles and any articles not in the English language.

**Figure 1:**
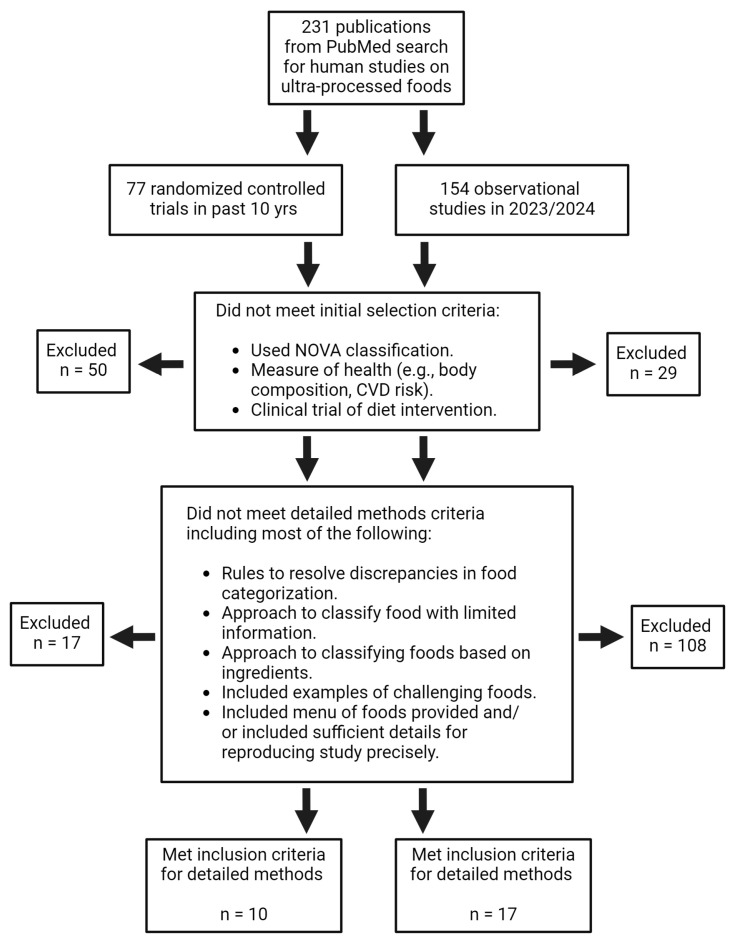
Flow chart of PubMed search strategy for clinical trials using UPF categorization methods.

Randomized controlled trials (RCTs) were restricted to the previous 10 years and observational studies were restricted to 2023 and 2024. The exact search terms used to find RCTs and observational articles are detailed in Tables S1 and S2. The number of articles found and excluded are shown in Figure 1.

Inclusion criteria: The methodology for each article found in the searches was reviewed and studies were included if they described classifying foods into categories based on the degree of processing using the NOVA method. The selected articles included a measure of health (e.g., body composition, CVD risk). Articles were also evaluated for the level of detail in their methods of categorizing UPFs. Highly detailed articles from the list are included in Table 2 and Table 3. Articles were considered to be highly detailed in their methods of categorizing UPFs if they included most of the following elements: (1) the classification rules used to resolve food classification discrepancies, (2) a method to classify food items with limited or missing information (e.g., foods prepared at restaurants), (3) a method to classify foods as UPFs based from specific ingredients, (4) a list and discussion of specific challenging examples, and (5) a full or sample menu of food provided in the study or sufficient details to reproduce the methods.

Exclusion criteria: We excluded articles that were not related to classifying food by processing level (i.e., articles related to neurological processing), articles using food classification systems other than NOVA, and articles that were limited to a specific food (i.e., processed meats) instead of food processing in general. Articles involving animal subjects were also excluded.

## 3. UPFs and the Gut Microbiome

### 3.1. Analysis of the Gut Microbiome in Studies with a Focus on UPF Classification

Diet has been shown to quickly modify the gut microbiome, and the gut microbiota play an active role in human health, ranging from metabolism to immunity to disease susceptibility [12,13,14], and the microbiome has potential use in predicting the risk, progression, and severity of disease [15]. Recent human studies that have examined the associations between UPFs and the gut microbiome, separate from food that is part of a Western diet, are outlined in Table 1, and their additional study characteristics can be viewed in Table S3.

These four observational studies use the NOVA classification system to identify UPFs (two using FFQs and two using 24 h dietary recalls); however, each uses a unique system of comparing UPFs within their populations. For example, Cuevas-Sierra et al. [16] compare “high” UPF consumption vs. “low” UPF consumption (>5 servings/day and <3 servings/day, respectively), while Atzeni et al. [17] compare tertiles of proportions (percentage of total daily calorie intake) of UPF consumption (where low = first tertile, medium = second tertile, and high = third tertile). This prompted us to examine the individualized methods within the studies that used NOVA to categorize their food items. While Atzeni et al. and Fernandes et al. provide no details beyond the general references for using NOVA, Cuevas-Sierra et al. included a supplemental list of all foods classified as UPFs in their FFQ and García-Vega et al. describe details of their process for categorizing foods from their 24 h dietary recalls [16,17,18,19]. García-Vega et al. classified all food items as either ultra-processed or not ultra-processed. Of the 14,375 food items reported in the study, 93% (13,358 food items) were easily categorized and 7% could be classified in either group (e.g., Colombian food called arepa, a grilled patty of soaked ground kernels of corn or corn flour, is equally common to be prepared at home or bought in industrialized form). In the case of uncertain foods, the authors considered items to be UPFs. A sensitivity analysis was performed by evaluating the effect of classifying these foods as not ultra-processed, which did not affect the results [19]. This highlights the heterogeneity of methods used to collect and categorize food information, which makes reproducing studies difficult, but suggests that the conclusions from multiple studies are robust despite the subset of difficult-to-classify food items.

All four UPF studies analyzed the gut microbiome using a single fecal sample collected per individual and 16S rRNA sequencing of various hypervariable regions. Diversity matrices are often included in microbiome studies, and it has been shown that diversity in the gut can be affected by both diet and body size, though the relevance of these matrices in deciphering the interplay between diet, gut microbiome, and health remain unclear [20]. Only one of the four UPF studies showed a difference in alpha diversity, while none of the studies observed significant differences in beta diversity. Cuevas-Sierra et al. revealed that men consuming >5 servings/day of UPFs had lower richness compared to men consuming <3 servings/day; however, no differences were seen in women or the whole population [16]. This study indicates that the associations between UPFs and gut microbiota may be sex-specific; however, other studies adjusting their analysis for sex did not observe differences [17,19]. Two studies identified increased *Prevotella* spp. [17,19] and two studies identified decreased *Lachnospira* spp. [16,19] and *Ruminococcus* spp. [18,19] with increased UPF consumption. Interestingly, García-Vega et al. showed that *Prevotella* spp. (*P. copri* and *P. melaninogenica*) were associated with increased intake of animal-derived foods, while *Ruminococcus* spp. (*R. bromii* and *R. albus*) and a *Lachnospira* sp. were positively associated with intake of plant-derived food groups [19]. The Firmicutes/Bacteroidetes (F:B) ratios, which sometimes correlate with obesity, disease states, or dietary patterns [21,22,23], were not significantly correlated with UPF consumption in three of the studies [16,17,18]. 

Although there are only a few studies in Table 1, there are numerous studies reporting changes to the gut microbiome in response to dietary changes that would affect UPF consumption, even if UPF classification was not a focus of each study. For example, individuals switching from a Western diet to a Mediterranean diet would most likely have a significant drop in their UPF consumption, and the gut microbiome changes in those studies likely overlap with the changes that might be seen on a diet focused on reducing NOVA 4 foods [24,25,26,27,28,29,30]. The potentially significant differences in specific UPFs and UPF ingredients between the various dietary intervention designs is worth consideration, and additional studies with a specific focus on UPFs and the gut microbiome are needed. UPFs are often associated with negative health outcomes. Some general health outcomes of the four UPF studies are discussed here, and additional information can be found in Table S3. The study by Atzeni et al. was a substudy of the PREDIMED-Plus trial, which analyzed the habitual diet of older volunteers with overweight/obesity and metabolic syndrome. They found that while UPF consumption was positively associated with a lower Mediterranean diet adherence score, there were no significant differences in insulin, cholesterol, triglycerides, body weight, or body mass index (BMI) between those consuming the lowest (7.177 ± 2.349% kcal/day) and highest (21.4 ± 5.0% kcal/day) amounts of UPFs [17]. Cuevas-Sierra et al. also reported on the habitual diet of adults in the Obekit trial but analyzed men and women separately from the whole population. They discovered several sex-dependent differences in their cohort; women consuming >5 servings/day of UPFs reported more cases of depression and anxiety and higher weight and hip circumference. Meanwhile, men consuming >5 servings/day of UPFs had decreased levels of high-density lipoprotein (HDL) cholesterol and higher weight and BMI [16]. The study by Fernandes et al., which only included women, found that UPF consumption was associated with leptin resistance after adjusting for fat mass, but no other anthropometric or clinical variables [18]. Lastly, the study by Garcia-Vega et al. recruited individuals in equal proportions by city of origin, sex, age range, BMI, and socioeconomic status, but did not compare these variables to UPF intake, nor did they measure other metabolites or other clinical variables [19]. Given that there are only three studies here that examined clinical outcomes pertaining to UPF intake, no trends can reasonably be drawn. Additional studies incorporating measurements of food processing level, gut microbiome, and clinical data are needed before we can start to piece together the definitive effects of UPFs on the gut microbiome and health.

These studies have several strengths and limitations. The strengths include the fact that statistical analyses of these studies were adjusted for several potential confounding factors, including age, BMI, sex, and others, where applicable. The sample sizes were sufficient, with the smallest sample being 59 women [18] and the largest being 645 individuals [17]. A limitation of cross-sectional observational studies is the inability to infer causal relationships. The use of 16s rRNA sequencing analysis often limits taxonomic profiling to genus-level data. Although widely accepted, both FFQs and 24 h dietary recalls are limited in their usefulness to assess UPFs due to generalized food categories and limited sampling frequency. While two of the four studies used dietary quality indices (Healthy Eating Index and Colombian Food-Based Dietary Guidelines [19] and Mediterranean Diet Score [16]), neither adjusted for these in their statistical analysis, so the influence of diet quality cannot be ruled out as a confounder. Lastly, the differences in how UPFs are categorized and compared within each study make comparing the data between studies challenging. Changing the cut-off values for servings per day or tertiles of UPFs may change the study results. Although there are few studies that have specifically examined the associations between UPFs and the gut microbiome, these are fairly detailed studies, and we expect to see data from several more observational and randomized controlled trials (i.e., Capra et al. [31]) within the next decade. Additional information can be gained from studies that were not specifically identified as being UPF studies, as is described below. 

**Table 1:** Methods and outcomes of clinical studies examining UPFs and the gut microbiome.

References	Gut Microbiome Collection Method and Frequency		Alpha Diversity	Beta Diversity	Microbiome Sequencing Analysis: Bacterial Composition Changes in Relation to UPFs		Composition Changes Related to Specific UPFs
					Increase ↑	Decrease ↓	
Atzeni, 2022 [17]	One stool sample collected by volunteers at home and frozen	METHODS	Chao1, Shannon, and Simpson indices analyzed with one-way ANOVA.	Euclidean distance analyzed by PERMANOVA.	16S rRNA analysis of the V4 variable region using Novaseq		No significant differences between bacterial taxa and UPF item categories.
		RESULTS	No significant differences.	No significant differences.	Positive association between *Alloprevotella* spp. (*p* = 0.041) and *Sutterella* spp. (*p* = 0.116) vs. tertile 2. Positive association between *Alloprevotella* spp. (*p* = 0.065), *Negativibacillus* spp. (*p* = 0.096), and *Prevotella* spp. (*p* = 0.116) vs. tertile 3.		
Cuevas-Sierra, 2021 [16]	One fecal sample self-collected by volunteer using OMNIgene. GUT kits from DNA Genotek (Ottawa, ON, Canada)	METHODS	Chao1 and Shannon indices analyzed using a paired non-parametric test.	Bray–Curtis index analyzed using PERMANOVA test.	16S rRNA analysis of the V3–V4 variable regions using MiSeq		Women: dairy and pizza positively correlated with Actinobacteria (*p* < 0.05), and pizza positively correlated with *Bifidobacterium* spp. (*p* < 0.05) Men: meat positively correlated with Bacteroidetes (*p* < 0.05)
		RESULTS	Men consuming >5 servings/day of UPFs showed lower richness compared to men consuming <3 servings/day (observed *p* = 0.03, Shannon *p* = 0.01, Chao1 *p* = 0.04), yet no differences in women or whole population.	No significant differences.	Whole population: *Gemmiger* spp. (*p* < 0.001), *Granulicatella* spp. (*p* < 0.001), *Parabacteroides* spp. (*p* < 0.001), *Shigella* spp. (*p* < 0.001), *Bifidobacterium* spp. (*p* < 0.001), *Anaerofilum* spp. (*p* = 0.001), *Cc_115* spp. (*p* = 0.007), *Oxalobacter* spp. (*p* = 0.008), *Collinsella* spp. (*p* = 0.008) Women: *Acidaminococcus* spp. (*p* < 0.001), *Butyrivibrio* spp. (*p* < 0.001), *Gemmiger* spp. (*p* < 0.001), *Shigella* spp. (*p* < 0.001), *Anaerofilum* spp. (*p* = 0.001), *Parabacteroides* spp. (*p* = 0.002), *Bifidobacterium* spp. (*p* = 0.006) Men: *Granullicatella* spp. (*p* < 0.001), *Blautia* spp. (*p* = 0.002)	Whole population: *Lachnospira* spp. (*p* = 0.003), *Roseburia* spp. (*p* = 0.003) Women: *Melainabacter* spp. (*p* = 0.002), *Lachnospira* spp. (*p* = 0.003) Men: *Anaerostipes* spp. (*p* < 0.001)	
Fernandes, 2023 [18]	One fecal sample collected at home; one aliquot was stored in a tube containing 3.5 mL of guanidine for genomic DNA conservation	METHODS	Chao1, Shannon, Simpson, and Observed Species indices analyzed using Pearson’s correlation coefficients.	N/A	16S rRNA analysis of the V2–V4 + V6–V9 (excluding V1 and V5) variable regions using Ion Torrent Personal Genome Machine™		N/A
		RESULTS	No associations between food processing level and alpha diversity.	N/A	*Clostridium butyricum*, *Odoribacter splanchnicus*, *Barnesiella intestinihominis* *Alistipes onderdonkii*, *Alistipes indistinctus*,	*Ruminococcus* sp., *[Ruminococcus] gnavus*, *Bacteroides vulgatus*, *Bacteroides plebeius*	
García-Vega, 2020 [19]	One fecal sample self-collected by volunteer at home, refrigerated, and brought to the lab within 12 h	METHODS	Estimates calculated with BiodiversityR 2.11. Shannon and Shannon evenness (Jevenness) indices calculated using Vegan 2.5 and tested with ANOVA.	Estimates calculated with GUniFrac 1.1 and tree-based UniFrac distances tested with PERMANOVA.	16S rRNA analysis of the V4 variable region using MiSeq		OTUs from *Oscillospira* sp., unclassified *Ruminococcaceae*, *Ruminococcus* sp., *Lachnospira* sp. positively associated with intake of plant-derived food groups, rich in dietary fiber; *Bifidobacterium adolescentis* associated with plant-derived food groups; bile-tolerant *Bilophila* sp., *Prevotella copri*, and the opportunistic pathogen *Prevotella melaninogenica* were associated with increased intake of animal-derived foods
		RESULTS	Higher in females than males (Shannon, *p* = 0.046), higher in middle-aged than younger individuals (Shannon, *p* = 0.012). No significant association between diet quality (including UPF intake) and alpha diversity.	Differences according to participants’ city of origin (*p* = 0.001), sex (*p* = 0.001), socioeconomic level (*p* = 0.024) and BMI (*p* = 0.002). No significant association between diet quality (including UPF intake) and beta diversity.	*Bifidobacterium adolescentis*, *Prevotella melaninogenica*, *Subdoligranulum variabile*, *Veillonella dispar*, *Ruminococcus* sp., *Bilophila* sp., *Oscillospira* spp.	*Prevotella copri*, *Clostridium hathewayi*, *Ruminococcaceae* unclassified sp., *Gemella* sp., *Lachnospira* sp., *Oscillospira* spp.	

Abbreviations: UPF = ultra-processed food; BMI = body mass index; OTUs = operational taxonomic units; PERMANOVA = permutational multivariate analysis of variance; N/A = not applicable.

### 3.2. Plant-Based Diets: Health Conseqences and Effects on the Gut Microbiome

While the term “ultra-processed foods” may be relatively new to the nutrition field, researchers, clinicians, and nutritionists have been studying these types of food categories, along with the benefits of whole-food diets and anti-inflammatory diets, for many years. For example, foods high in added sugars, salts, and food additives, which very often are classified as NOVA 4 ultra-processed foods, are generally recognized as foods that should be limited in several healthy diets such as the Mediterranean diet, the Dietary Approaches to Stop Hypertension (DASH) diet, and the World Cancer Research Fund and the American Institute for Cancer Research (WCRF/AICR) dietary recommendations, among others. Multiple studies have been conducted in cohorts who follow a healthy plant-based diet. A recent cross-sectional analysis of baseline data from 596 participants enrolled in the Mediterranean healthy Eating, Aging and Lifestyle (MEAL) study showed that high UPF consumption correlated with low adherence to the Mediterranean diet and higher odds of having depressive symptoms [32]. The DASH diet, which emphasizes intake of fruits and vegetables, whole grains, and low-fat dairy, is one of the most widely prescribed diets for reducing blood pressure and risk of CVD [33]. A 12-week randomized low-calorie DASH diet intervention in adults with obesity (*N* = 120) resulted in a decreased F:B ratio compared to an alternative low-calorie diet, and reduced LPS levels compared to the control group (not calorie-restricted, counseled to plan meals, to minimize over-eating and high-fat foods, and to exercise to achieve weight loss) [34]. The WCRF/AICR diet is considered an anti-inflammatory diet and limits the consumption of fast foods and processed foods. Colorectal adenoma patients (*n* = 97) and healthy volunteers (*n* = 54) who adhered to the WCRF/AICR diet had reduced relative abundance of unidentified *Enterobacteriaceae* spp. compared to subjects with low adherence. Interestingly, restricting fast-food intake was associated with high *Bacteroidaceae* and *Bacteroides* spp. abundance, and reduced inflammatory biomarker immunoglobulin G (IgG) levels in men, while limiting sugary drinks was associated with reduced *Lachnospiraceae* [35]. The population-based *Milieu Intérieur* study of 862 healthy French adults showed that bacterial diversity (Simpson’s index) was negatively associated with food items that are generally recommended for limited consumption, such as fried foods, sodas or sugar-sweetened beverages, ready-cooked meals, and desserts. Assessment of the relative abundance of the gut microbiome using 16S rRNA sequencing and multivariate association with linear models found that *Streptococcus parasanguinis* and *Prevotella oulorum* negatively correlated with sodas or sugary drinks, while there was a trend toward positive associations between *Blautia luti* (*p* = 0.06) and *Ruminococcus gauvreauii* (*p* = 0.07) and sweets, and *Bifidobacterium adolescentis* (*p* = 0.07) and fatty sweet products [36]. These healthy plant-based dietary interventions are a few examples of many studies that can be used to make general hypotheses for the effects of UPFs on the gut microbiome and health outcomes, and are useful for designing future studies.

In contrast, the Western diet, which is a diet prevalent in most industrialized nations, is high in saturated fats, salt, animal proteins, and UPFs. Studies of the health impact of UPFs overlap with studies of the impact of a Western diet, as a Western diet typically includes a high proportion of UPFs [1]. The effects of a Western diet on the human microbiome have been reviewed elsewhere [1,2,8,37].

Upon review of the numerous published studies on the gut microbiome and dietary interventions, the complexity of the microbiome is beyond question. There are some bacterial modifications that recur in multiple studies as associations with dietary change (i.e., increased *Roseburia* spp., decreased *Ruminococcus* spp., and decreased Firmicutes/Bacteriodes ratio in response to a Mediterranean diet). However, there are many more examples of species changes that are only seen in one or a few studies to date. This emphasizes that we do not yet know which species might be critically important, or which have substitutes that are equally functional in a particular pathway, or if the bacterial community composition (not specific species) and non-bacterial microbiota might be the key. We refer the reader to other comprehensive reviews on this topic and look forward to future scientific advances in the area of gut microbiome changes with specific dietary components.

### 3.3. Fast-Food Meals: Effects on Gut Microbiota and Metabolites

According to the Centers for Disease Control and Prevention (CDC), during 2013–2016, approximately 37% of adults consumed fast food each day, reported as “restaurant fast food/pizza” in 24 h dietary recalls collected by the National Health and Nutrition Examination Survey (NHANES) study [38]. Fast-food meals are categorized as UPFs, are typically high in saturated fat, sugar, salt, and calories, are a usual part of the Western diet, and have been shown to modify the gut microbiome. A randomized cross-over trial of ten healthy subjects consuming a fast-food (i.e., burgers and fries) or Mediterranean diet for 4 days each, with a 4-day washout period between diets, showed increases in bile-tolerant bacteria, including *Collinsella* spp., *Parabacteroides* spp., and *Bilophila wadsworthia*, and decreased fiber-fermenting bacteria, including *Lachnospiraceae* spp. and *Butyricicoccus* spp., after the fast-food diet [39]. A prospective observational study of 25 healthy individuals provided fast-food meal options (i.e., burgers, French fries, chicken nuggets, pizza, pasta, beverages, and dessert/ice cream) from two major international franchises, a local pizza kitchen, and non-diet sodas ad libitum for two hours did not find differences in fecal bacterial diversity (Shannon and Simpson indices) and communities from before and the day after the fast-food binge. Indeed, changes in gut microbiota might not be expected to be measurable in fecal specimens in such a short period of time. However, there were several significant changes in serum total and individual primary and secondary bile acids [40]. Although both of these studies were very short (1–4 days), they provide evidence that fast-food meals may impact the gut environment compared to both baseline and healthy (Mediterranean) diets.

### 3.4. UPF Meals/Supplements with High Nutritional Value: Effect on the Gut Microbiota and Metabolites

The long shelf life and stability of ready-to-consume rations make them advantageous for providing nourishment to the hundreds of thousands of U.S. military personnel and civilians each year involved in natural disasters. These meals are similar to the average American diet in micronutrient proportions and fiber density and are fortified to comply with micronutrient requirements from the U.S. military dietary intake reference [41,42]; these meals are also highly processed with no fresh foods [43]. These ready-to-eat meals comprise an entrée, a starch, a spread (cheese, peanut butter, jam/jelly), a dessert and/or a snack, a beverage powder, instant coffee or tea, and chewing gum, and average 1250 kcal. For example, one menu may include meatballs in marinara sauce, blueberry cobbler, a chocolate chip cookie, a jalapeno cheddar cheese spread, Italian bread sticks, a teriyaki meat snack stick, an electrolyte beverage powder, and chewing gum. Except for the beverages, the entire meal is ready to eat and can be consumed cold or heated by submerging in hot water or using a flameless ration heating device, which is also provided with meals, along with other toiletries (i.e., hand and body wipe, toilet tissue). The meals have an average shelf life of three years at 80 °F, and shelf life can be expanded through the use of cold storage facilities prior to distribution [44]. A secondary analysis of a controlled trial examining healthy subjects randomized to eat their usual diet (*n* = 30) or a Meal, Ready-to-Eat™ (Ameriqual Packaging, Evansville, IN, USA, *n* = 30) military ration diet for 21 days found no significant changes in diversity (observed OTUs, Shannon, Bray–Curtis), but some significantly increased (*Ruminococcus* spp., *Veillonella* spp., *Clostridium* spp. and *Sutterella* spp.) and decreased (*Leuconostoc* spp., *Lactococcus* spp. and *Lactobacillus* spp.) relative abundance of bacteria following the Meal, Ready-to-Eat diet [41]. The same group found a significant impact on the fecal metabolome, including increased concentrations of multiple dipeptides and long-chain saturated fatty acids, while plant-derived compounds and bile acid metabolism were decreased in the Meal, Ready-to-Eat™ group. The differences in diet groups were driven by several primary and secondary bile acids and caffeine-derived metabolites, which correlated with changes in the relative abundance of *Megasphaera* spp., *Clostridium* spp., *Sutterella* spp., *Prevotella* spp., and *Collinsella* spp. [45]. 

Another situation when long-shelf-life convenient foods might be consumed in association with otherwise health-conscious activity, beyond rations for the military forces, is food consumption by athletes during training or competitions. Similarly, sports nutrition supplements, which contain food additives such as artificial sweeteners, emulsifiers, preservatives, and acidity regulators, are used by athletes to improve performance and recover faster. A recent review by Alvarez-Herms, Gonzalez-Benito and Odriozola [46] outlined the gastrointestinal issues experienced by some athletes, which they explain are directly related to loss of gut equilibrium, microbiota dysbiosis, and leaky gut, and may be due in part to the elevated intake of UPFs. Although meal rations and athletic supplements are often high in nutritional value, they are also considered UPFs, and the long-term effects of eating these types of foods are not yet clear.

### 3.5. Food Additives

There is a wide range of food additives in UPFs, and given space limitations, we have chosen to not include an in-depth discussion on food additives. Although not our focus, food additives must be considered when investigating the effects of UPFs because most UPFs contain food additives, and these have been proposed to contribute to the deleterious effects of UPFs on health [7,47]. A large (*N* = 92,000) prospective study of the French NutriNet-Santé cohort found that higher intake of emulsifier mono- and diglycerides of fatty acids (E471) were associated with increased risk of overall cancer, breast cancer, and prostate cancer, while higher intake of carrageenans (E407 and E407a) was associated with increased risk of breast cancer [48]. One mechanistic route by which food additives may affect health is through the gut microbiome, as has been shown in clinical studies using artificial sweeteners [49], guar gum [50], and carboxymethylcellulose [51]. In contrast, some clinical studies of food additives have shown no or minimal effects on the gut microbiota, including studies using tart cherry concentrate [52], sugar beet pectin [53], and artificial sweeteners [54,55,56,57], or have been even shown to improve health [58]. In a randomized cross-over exploratory analysis, Berding et al. investigated the effects of polydextrose, a dietary fiber, on cognitive performance and acute stress response in eighteen women over 4 weeks. Polydextrose treatment resulted in increased abundance of *Ruminiclostridium* 5 and a modest improvement in cognitive performance (measured by a decrease in errors made in the Intra-/Extra-Dimensional Set Shift task and higher number of correct responses and rejections in the Rapid Visual Information Processing task) compared to placebo [58]. These studies highlight the huge number of different food additives and the complexity of the effects of many food additives on human health. Clearly, not all food additives, just like UPFs, should be grouped together.

## 4. Methods for Classifying UPFs

The methods used in dietary studies vary both by collection tool (e.g., FFQs, 24 h dietary recalls, food records) and frequency, and each method has strengths and limitations for identifying UPFs. While FFQs lack the ability for researchers to categorize foods based on ingredients, they can offer a more systematic way of categorizing foods using computer codes. However, not all mixed-dish foods or packaged items entered into nutrition databases can be broken down by ingredients, and ingredients can vary greatly between food brands. Thus, research teams often need to come to some consensus to make decisions on how to categorize certain foods for their studies. One of the current challenges with using the NOVA classification system for categorizing UPFs is the ambiguity of how to best classify some methods of food processing and ingredients. NOVA, like most other food classification systems, considers the extent (how different the food is from the core unprocessed food), nature (e.g., use of food additives or changing food properties), purpose (e.g., preservation, appearance, texture, etc.), and location (homemade vs. commercially made) of processing, which can be hard to define, especially when information is unavailable to the investigator [10,59]. An article published in 2022 featured a discussion related to the conceptualization of processed foods and challenges for communication amongst 27 professionals in the fields of nutrition, food technology, policy making, industry, and civil society in an online discussion group, who agreed that consensus is important but challenging [60]. In 2023, an interdisciplinary, multi-stakeholder workshop of professionals in government, academia, and industry convened to develop a research roadmap of research priorities about processed food intake and health risk in U.S. populations. Discussion on the subjectivity in UPF classification and the heterogeneity of foods/beverages classified as such warrant the need for advances in classification and food intake assessments for researchers [61].

Many of the studies that we reviewed in our initial search were excluded from our tables due to a lack of sufficient details in their food classification system methods, instead only citing the original NOVA definitions. Lack of detail in classification methods makes comparing the data between studies even more challenging. With a goal in the field to come closer to a mechanistic understanding of UPFs’ effect on health, we asked the following questions: (1) how many UPF studies provided very detailed methods? and (2) how much uncertainty might result from the missing details? The second half of this review will discuss our analysis of recent UPF clinical studies and the findings from a representative sampling of clinical randomized controlled trials and observational studies that include detailed methods for using the NOVA classification system. We sought to provide useful information for those designing and interpreting UPF dietary clinical trials.

### 4.1. Randomized Controlled Trials

Out of the 77 RCTs identified from our initial PubMed search, 10 studies with the most detailed methods were included in our analysis (Figure 1). The discussion in this section will refer primarily to the study methods described in Table 2, of which their additional study characteristics can be viewed in Table S4. To date, only one study (resulting in at least two publications) has been completed that compares a study-provided UPF diet to an unprocessed diet [62,63]; however, at least two additional studies are currently underway [31,64]. For the studies not providing a UPF diet, two used 24 h dietary recalls [65,66], three used FFQs [67,68,69], and one used food records [70] to collect food information. Generally, the RCT studies that include information on discrepancy resolution describe either a majority-rule or consensus method between study team members about unclear food and beverage items [66,67,69,70]. More RCT studies are needed with a focus on UPFs to bring us closer to identifying causality and not merely associations of these foods with human health.

RCT designed studies have many benefits, including that they (1) can control for specific variables, (2) generally require smaller sample sizes to obtain meaningful results, (3) have higher granularity in the data collected, (4) often collect more comprehensive patient health information (e.g., stool, blood, and urine metabolomics, immune analyses, gut microbiome, host genetics, etc.), (5) have better knowledge of meta-data (e.g., physical activity), (6) more easily allow for comparison between studies, and (7) can be designed to dissect possible causality in humans to inform the design of animal models. Of note, but outside the scope of this review, there have been several studies that examined specific types of food processing such as processed meats or milk alternatives, which are also valuable to understand the differences in processed versus ultra-processed foods [71,72,73]. The limitations for these RCT studies include that (1) they often only follow individuals for a short period of time (days to weeks), which does not allow researchers to assess the stability of the study findings and limits the generalizability of the data; (2) they mostly rely on self-reporting of information from volunteers (e.g., 24 h dietary recalls or food records); (3) they are time-consuming and expensive to conduct; and (4) the lower number of participants can limit the statistical power of the results. Below, we discuss RCT studies that provide food (Section 4.1.1) or that use food collection methods (Section 4.1.2) to examine the effects of UPFs on diet and health. We compare their detailed methods for classifying UPFs and their sensitivity analyses, and summarize their findings of the effect of UPFs on health.

#### 4.1.1. RCTs That Provided All Food

Providing food is beneficial in that it eliminates some of the ambiguity surrounding food classifications, such as whether food is homemade or commercially/industrially made, and can limit the burden of work for the volunteer, who may not need to report details or amounts of food consumed. Providing food also reduces the need for volunteers to self-report their diet, which can be prone to error [74]. However, RCTs, especially those that provide food (such as the three interventions described below), are difficult to conduct, mostly limited by the cost to conduct the study and/or provide food. Very few RCTs comparing UPF vs. non-UPF diets, without some additional dietary component (i.e., Western diet or Mediterranean diet), have been conducted. However, these studies have the added benefit of formulating comparable diets and considering other confounding dietary factors. For example, the study by Hall et al. designed meals to be matched for calories, energy density, macronutrients, sugar, sodium, and fiber intake. In order to match for fiber, the UPF diet required fiber (NutriSource) supplementation in 20 out of the 21 meals [62]. The diets designed by Capra et al. controlled for fiber, added sugars, mono- and poly-unsaturated fats, saturated fat, sodium, glycemic index and load, and overall diet quality via the Healthy Eating Index (HEI)-2015 [31]. Similarly, the diets designed by Rego et al. were matched in nutrients, fiber, added sugars, mono- and poly-unsaturated fats, saturated fat, sodium, glycemic index, and overall diet quality via the HEI-2015 [64]. Because the NOVA 4 category can include any type of food and can range from a food with no nutritional value to a food with perhaps only one additive and significant nutrients, controlling for other dietary factors, as described above, is vital for teasing apart the effects of different components of a diet high in UPFs to better understand which components are biologically most important for health.

RCT studies presenting their full [62,63] or partial [31,64] meal choices show a high level of transparency for categorizing foods. These also allow for the assessment and consideration of exact nutrients and food additives that are otherwise estimated or unknown. For example, Capra et al. counted the number of food additives included in their menus and found that the most commonly consumed food additives (eaten ≥10 times per week) were high-fructose corn syrup, soy lecithin, citric acid, sodium citrate, annatto color, artificial flavors, and sorbic acid [31]. These studies also allow for a critical comparison of UPF categorization. Below are some examples of foods that often require discrepancy discussions amongst study team members that are included in RCT studies providing food and illustrate the differences between study designs and classification. Some of the food items classified as non-UPFs below are classified as NOVA 4 in other studies.

- Cheeses:
  - Hall et al. included parmesan cheese (Roseli, Rosemont, IL, USA), American cheese (Glenview Farms, Rosemont, IL, USA), provolone cheese (Roseli, Rosemont, IL, USA), Monterey Jack cheese (Glenview Farms, Rosemont, IL, USA), cream cheese (Philadelphia, Chicago, IL, USA), and shredded cheddar and Monterey Jack cheese (Glenview Farms, Rosemont, IL, USA) on their UPF menu, while no cheese was included on their non-UPF menu [62].
  - Capra et al. listed parmesan, cheddar, and American cheese as examples on their UPF menu, while parmesan and cheddar cheese were also featured on their non-UPF menu [31].
  - Rego et al. showed Kraft (Northfield, IL, USA) American Cheese on their sample UPF menu, and Kroger (Cincinnati, OH, USA) natural cheddar cheese on their non-UPF menu [64].
- Bread:
  - Hall et al. included white bread (Ottenberg, Bethesda, MD, USA), croissants (Chef Pierre, Oatbrook Terrace, IL, USA), English muffins (Sara Lee, Downers Grove, IL, USA), hoagie rolls (Ottenberg, Bethesda, MD, USA), and plain bagels (Lender’s, Horsham, PA, USA) on their UPF menu, while the non-UPF menu featured other types of grains (rice, bulgar, oatmeal, quinoa, farro, etc.) [62].
  - Capra et al. listed commercial white buns and commercial whole-wheat buns as examples on their UPF menu, while homemade bread was included on their non-UPF menu [31].
  - Rego et al. showed Wonder bread (Thomasville, GA, USA) as an example on their UPF menu, while homemade bread was part of their non-UPF menu [64].
- Sweet snacks:
  - Hall et al. included blueberry muffins (Otis Spunkmeyer, San Leandro, CA, USA), Fig Newtons (Nabisco, East Hanover, NJ, USA), honey buns (Little Debbie, Collegedale, TN, USA), Graham crackers (Nabisco, East Hanover, NJ, USA), applesauce (Lucky Leaf, Peach Glen, PA, USA), oatmeal raisin cookies (Otis Spunkmeyer, San Leandro, CA, USA), and shortbread cookies (Keebler, Battle Creek, MI, USA) on their UPF menu, while only fresh, frozen (without added sugar), or dried (raisins) fruits were provided in the non-UPF diet [62].
  - Capra et al. listed Skittles (Mars Wrigley, Chicago, IL, USA) and Chips Ahoy! Cookies (Nabisco, East Hanover, NJ, USA) as snack examples in their UPF diet, and natural fruit licorice candy in their non-UPF diet [31].
  - Rego et al. also showed Skittles (Mars Wrigley, Chicago, IL, USA) and Chips Ahoy Cookies (Nabisco, East Hanover, NJ, USA), along with Pop Tarts (Kellanova, Battle Creek, MI, USA), Keebler (Battle Creek, MI, USA) Old Fashioned Sugar Cookies, and Welch’s (Concord, MA, USA) Fruit Snacks in their UPF diet, while homemade sugar cookies, homemade banana muffins, and Panda (Vaajakoski, Finland) Natural Raspberry Licorice were included in the non-UPF diet [64].
- Savory snacks:
  - Hall et al. included potato chips (Lay’s, Plano, TX, USA), baked potato chips (Lay’s, Plano, TX, USA), baked Cheetos (Frito-Lay, Plano, TX, USA), tortilla chips (Tostitos, Dallas, TX, USA), dry roasted peanuts (Planters, Austin, MN, USA), cheese and peanut butter sandwich crackers (Keebler, Battle Creek, MI, USA), and Goldfish crackers (Pepperidge Farm, Norwalk, CT, USA) in their UPF diet, while savory snacks were replaced with raw nuts (almonds, walnuts) in the non-UPF diet [62].
  - Capra et al. listed Ritz Crackers (Nabisco, East Hanover, NJ, USA) in their UPF diet, compared to Good Thins rice crackers (Mondelez International, East Hanover, NJ, USA) in their non-UPF diet [31].
  - Rego et al. showed plain Pringles (Kellanova, Battle Creek, MI, USA) and Ritz Crackers (Nabisco, East Hanover, NJ, USA) in their UPF diet, compared to Cape Cod Kettle Cooked Chips (Charlotte, NC, USA) and Good Thins rice crackers (Mondelez International, East Hanover, NJ, USA) in the non-UPF diet [64].

**Table 2:** Methods of clinical randomized controlled trials using the NOVA system to categorize UPFs.

References	Food Collection Method and Frequency	Nutritional Program for Data Entry	Classification Method	Discrepancy Resolution	Examples of ‘Difficult’ Food Categorization/UPF Brands Used in Menus/Comments
Capra, 2024 [31]	N/A (Plan to collect three 24 h dietary recalls of habitual diet, then study food will be provided)	NDS-R 2022, Nutrition Coordinating Center, University of Minnesota	Nutrition label for each food item was used to classify menu foods manually using NOVA. Recipes for non-UPFs were developed to provide alternatives for commercial items like bread. Ingredient and menu examples provided in original article.	Not described	UPF breakfast menu contains Eggo waffles vs. non-UPF menu contains homemade waffles UPF snack menu contains apple slices with peanut butter vs. non-UPF menu contains natural fruit licorice candy Most common food additives (eaten ≥ 10 times per week) in the UPF menus: high-fructose corn syrup, soy lecithin, citric acid, sodium citrate, annatto color, artificial flavors, sorbic acid
Fagherazzi, 2021 [65]	Two 24 h recalls administered during the third and fifth appointments (6–8 and 12–14 weeks of intervention)	Microsoft Office Excel^®^ spreadsheet validated by Campos et al. [75]	Foods were classified according to NOVA and Dietary Guidelines for the Brazilian Population. When inadequate details provided, foods were categorized based on the typical form in which they are consumed.	Not described	Processed fruit juices and yogurts categorized as UPFs when brands were not provided
Fangupo, 2021 [67]	FFQ completed by parent on at least one of three occasions: 12, 24, and 60 months of age	N/A	Foods were classified based on the NOVA system. Product/recipe ingredients taken into consideration. Less straightforward items were disaggregated when able or discussed.	Consensus reached by researchers regarding how to disaggregate and categorize unclear foods	Categorized bacon, peanut butter, and cheese as NOVA 3 Categorized bread, commercial hummus, chocolate as NOVA 4 Items requiring disaggregation or discussion: porridge, canned fruits, pasta or tomato sauce, other fresh or canned fish, yogurt, Subway sandwich, kebabs or wraps, sushi, etc.
Gonzalez-Palacios, 2023 [68]	FFQ collected at baseline and 6 and 12 months	N/A	Specialized working group of experts in nutritional epidemiology and dieticians classified all FFQ items using NOVA. Supplementary Table S1 of original article shows classification of the 143 items in FFQ into each NOVA group, 36 of which were classified as UPFs. UPFs were further subdivided into six subgroups.	Not described	Coffee classified as NOVA 1, but decaffeinated coffee classified as NOVA 3 Items classified as NOVA 3: bacon or similar, homemade potato chips, homemade pastries, jams, dessert wine Items classified as NOVA 4: breakfast cereal, pastries or similar, chocolates and chocolate, cocoa powder
Hall, 2019 [62]	Study-designed diets provided for two weeks each (inpatient) without a washout period	ProNutra software (version 3.4, Viocare, Inc., Princeton, NJ, USA)	Food and beverages categorized according to NOVA. Detailed 7-day rotating menus with food brands provided in supplement.	Not described	UPF snack menu contains baked potato chips (Lay’s), dry roasted peanuts (Planters) and applesauce (Lucky Leaf) vs. non-UPF menus contain raisins (Monarch), fresh fruits, and raw nuts (Giant & Diamond)
Konieczna, 2021 [69]	FFQ collected at baseline, 6 and 12 months	N/A	Two dietitians independently classified all FFQ items using NOVA, then reviewed by nutritional epidemiologists.	Discrepancies in categorizations of food and drinks were discussed and consensus reached	The FFQ does not differentiate between plain, sweetened, or flavored yogurts and whole-grain cereals so they were grouped together as NOVA 1 Fruit juices, milkshakes, meatballs, hamburgers, and pizza, regardless of whether they are artisanal or industrial, were categorized as NOVA 4
O’Connor, 2023 [63] Refers to Hall, 2019 [62]	Study-designed diets provided for two weeks each (inpatient) without a washout period	ProNutra software (version 3.4, Viocare, Inc., Princeton, NJ)	Food and beverages categorized according to NOVA. Detailed 7-day rotating menus with food brands provided in supplement.	Not described	Refer to Hall, 2019 [62], above
Phillips, 2021 [70]	Smartphone app (myCircadianClock) used to record food and drink, and upload photos of food, drink, and medications daily	myCircadianClock entries categorized using Python scripts	Text entries classified by 4 independent reviewers. Food collected in German was classified by one reviewer due to language barriers. Some foods categorized by assumptions on base recipes and ingredients. Foods were assumed homemade unless stated otherwise or when processing was more common. Mixed dishes were classified to the highest NOVA group based on base recipe. Added new categories for beverages grouped into “Alcohol-containing drinks” (A), “Caffeinated drinks” (C), “Sweet drinks” (S), and “Other drinks” (D). Each drink could be assigned to multiple categories (e.g., soda Coca-Cola was ultra-processed, caffeinated, and sweet, abbreviated NOVA4-CS).	Consensus was reached for entries by at least 3 of 4 reviewers	Foods were assumed to be homemade with limited exceptions (i.e., chocolate-containing food and drinks, biscuits, toast and soft bread, croissants, pizza, burgers, plant-based drinks)
Rego, 2023 [64]	Study-designed diets provided (breakfast eaten in lab daily, remaining meals provided in portable cooler) Habitual diet determined using three 24 h dietary recalls	Open Food Facts app and NDS-R 2022, Nutrition Coordinating Center, University of Minnesota	Menus developed by a research dietician to meet UPF and other nutritional requirements and reviewed by a second dietician. Habitual diet UPF intake determined manually by trained evaluators using NDS-R output files and recall forms.	Not described	Breakfast cereal in UPF (Lucky Charms cereal) vs. non-UPF (Nature’s Path Organic Fruit Juice Corn Flakes Cereal) diet Snacks in UPF (Pringles, plain; Keebler Old Fashioned Sugar Cookie) vs. non-UPF (Cape Cod Kettle Cooked Chips; homemade sugar cookie) diet
Sneed, 2023 [66]	Three 24 h recalls each collected at baseline, 12, 24, and 36 months	NDS-R, Nutrition Coordinating Center, University of Minnesota	Some foods categorized by one expert coder to start, then six pairs of trained coders using NOVA and a set of decision rules adapted by the study team. Discrepancies resolved by defaulting to the higher processing level. Classification of mixed dishes were based on the processing level of the main ingredient contributing the highest calorie content and/or the methods used to prepare the food such as frying and not disaggregated.	Weekly meeting to discuss and resolve questions; study team made final decision to resolve coding discrepancies	Fast-food items typically considered minimally processed (e.g., 2% milk, apple slices, white rice, etc.) were further evaluated using ingredient label for industrial processing/food additives Difficulty distinguishing processed fruits (e.g., canned with added sugar) vs. ultra-processed fruits (e.g., canned with high-fructose corn syrup or sweeteners) Breads were generally classified as “industrial” and labeled as UPF unless explicitly noted as homemade or artisanal

Abbreviations: UPF = ultra-processed food; FFQ = food frequency questionnaire; NDS-R = Nutrition Data System for Research; N/A = not applicable.

#### 4.1.2. RCTs Using FFQ, Recalls, or Records

We will discuss six RCTs that utilized various dietary collection methods. The RCT studies in Table 2 utilizing FFQs provided detailed tables with food categorization [67,68,69]. The most common discrepancies from FFQ are either for categorizing mixed dishes, or when there is a lack of clarity for whether an item is homemade vs. commercially made. Unknown details for items can be handled in several ways, such as (1) assuming the highest category, (2) assuming the lowest category, or (3) disaggregating mixed dishes by their ingredients. For example, Konieczna et al. assumed fruit juices, milkshakes, meatballs, hamburgers, and pizza, which could be homemade or industrially made, to be the latter and categorized these as UPFs. However, yogurts and whole-grain cereals, which could be plain, sweetened, or flavored, were categorized as unprocessed or minimally processed foods [69]. Fangupo et al. listed all items on the FFQ that they considered as requiring disaggregation across categories (items that could not be categorized within only one of the four NOVA categories). Examples of these foods included canned fruits, potato salad, canned tomato sauce, jam, muffins or scones, yogurt, cream or sour cream, Subway sandwiches, kebabs or wraps, and sushi. Food was disaggregated using weighted criteria based on previous data or the relative market share in New Zealand, or using the researchers knowledge of New Zealander food purchasing and consumption habits [67]. While these studies provide a useful list of examples for food categorization, the typical person’s diet is not limited to a certain number or type of foods as captured on FFQs, so compromises must be made to fit the dietary data into the provided format.

Food records and 24 h dietary recalls provide the most specific and individualized accounts of food information. To our knowledge, no RCT studies using these methods have been designed to examine UPFs vs. non-UPFs in free-living individuals as the primary outcome; however, several have examined intake of UPFs as a secondary analysis to other dietary or health-related interventions [65,66,70]. Because these are secondary analyses, information relevant to categorizing UPFs may be missing in some cases. For example, when insufficient details were provided in 24 h dietary recall logs, Fagherazzi et al. classified foods according to the form in which the food was most usually consumed [65]. Philips et al. chose to be conservative with assigning the UPF category and categorized items as homemade, with few exceptions (e.g., pizza, burgers, plant-based drinks) [70]. Sneed et al. classified mixed dishes as homemade only if the phrase “from recipe or prepared from recipe” was listed in the food description; otherwise, foods were considered “ready-to-eat” [66]. Because food records and recalls typically require manual entry from the research team, there is an increased possibility of discrepancy in the way the food information is both entered and interpreted. To account for this, Sneed et al. adopted a rigorous training process for their NOVA coders to ensure the accuracy and consistency of the food categorization. Coders were also asked to rate the difficulty of categorizing each food independently on a scale of 1–4 (where 1 = “very easy” and 4 = “very hard”). The average self-rated difficulty was 1.4 (SD 0.55). Inter-rater reliability, based on the initial categorization attempts of coder pairs prior to final study team resolution, showed that 84.5% of foods had concordant categorizations, 11.2% had discordant categorizations, and 4.3% were initially coded as “I don’t know” by one or both coders. Food groups with the highest rates of discordance were fruits, condiments/spices, ready-to-eat foods, and grains. Validity of the study method’s approach was demonstrated using the finalized recall dataset to confirm that the NOVA categories aligned with expectations (e.g., UPFs generally had higher added-sugar-to-calorie and lower protein-to-calorie ratios and made up a high proportion of total daily calories) [66]. RCT studies analyzing UPF intake in free-living individuals offer insight to the most true-to-life situations of dietary and UPF consumption habits and are vital to our understanding of the effects of UPF on human health.

One of the most significant issues facing public health today is minimizing obesity and many obesity-related disorders due to the Western lifestyle (i.e., excess calorie consumption and lack of physical activity). Overweight/obesity has many potential negative health effects, and several RCTs included in our analysis suggest a link between UPFs and weight gain. In a controlled feeding trial, a high-UPF diet was associated with higher energy intake and increased weight compared to an unprocessed diet [62], and a secondary analysis revealed that 21 known and 9 unknown metabolites differed between diets [63]. Another study evaluating UPF intake in children found that higher added-sugar-to-calorie ratios and lower protein-to-calorie ratios correlated with UPF consumption [66]. UPF intake was associated with higher amounts of visceral fat in middle-aged (55–75 years) adults with overweight/obesity and metabolic syndrome [69]. Healthy diets such as the DASH and Mediterranean diet restrict the intake of UPFs [65,68]. In a Mediterranean diet intervention study, volunteers consuming the highest amounts of UPFs had increased risk for cardiometabolic risk factors (i.e., weight, BMI, waist circumference, and fasting glucose) compared to those consuming low amounts of UPFs [68]. Therefore, encouraging diets that are low in UPFs will likely play a major part in reducing obesity and its comorbidities. However, currently this is quite challenging as UPFs are ubiquitous, comprising up to 51% of the diet of children under 5 years [67], and they are generally less expensive than non-UPFs [31]. 

### 4.2. Observational Studies

Out of the 154 observational studies identified from our PubMed search, 17 were included in our analysis (Figure 1). This section will refer primarily to the methods described in Table 3, and additional study characteristics can be found in Table S5. More observational studies have been conducted compared to RCTs, as observational studies are often less resource-intensive, and the sharing of large datasets make them available for additional analyses beyond the primary analysis. Observational studies primarily rely on volunteers self-reporting food information, which is associated with errors (e.g., precision of food amount measurements, memory recall), may not capture other relevant health elements (e.g., physical activity, medications), cannot be used to compare UPF results precisely across studies, and cannot separate the different dietary elements of a Western-type or UPF diet such as salt, added sugar, and food additives. However, observational studies have the advantage of a generally larger sample size, longer time periods that data are collected, and can capture many different types of non-communicable diseases in a single study. For example, several studies in Table 3 utilize data from large cohort studies such as the European Prospective Investigation into Cancer and Nutrition (EPIC) and NHANES, which allows for analysis of thousands of individuals over many years [76,77,78,79,80].

Of the studies in Table 3, eight used FFQs [81,82,83,84,85,86,87,88], seven used 24 h dietary recalls [77,79,80,89,90,91,92], and two used a combination of methods (FFQs and food records) [76,78] to collect food information. Each of these methods have been validated for use in dietary research, but limitations exist that make food processing classification difficult in some cases. When there was ambiguity in the classification of foods, studies resolved these items by several methods: group discussion and consensus [83,86,90,91,92], discussion and defaulting to a lower level of processing [77,81,82,85,86,88,89], expanding discussion to an external group [87], and utilizing external resources such as grocery store labels [84,87,90,91]. As shown, some studies used a combination of these methods, while others did not describe how they worked to resolve discrepancies.

For instances of unclear NOVA categorization, it is necessary to have a set of study rules that researchers can refer to in order to make decisions. For example, Sullivan et al. had two researchers classify each food from an FFQ independently. When there were disagreements, the less-processed category was chosen [88]. Another research team in Australia collected food data using FFQs and determined the processing level for some difficult items such as bread, pasta, low-fat cheese, yogurt, and fruit juice using previous data collected from other Australian surveys, the National Nutrition Survey 1995 and the National Nutrition and Physical Activity Survey (NNPAS) 2011-12. When classification was still unclear, they used a conservative approach where the food was classified as non-UPF and disaggregated if it was a mixed dish [85]. A third group had two evaluators classify all foods from 24 h dietary recall data; then, a second set of two researchers verified the classifications. Decisions were made utilizing lists of ingredients from food packaging or company websites and discussion went on until consensus was reached [90]. These difficult cases highlight the lack of consensus on the application of the NOVA system. Some examples and how these were resolved by different studies are listed below:

- Bread:
  - Bonaccio et al. categorized all bread as NOVA 3 [81].
  - Cordova et al. assumed bakery breads from Italy and the UK to be NOVA 3 and commercial packaged bread to be NOVA 4 [76].
  - Houshialsadat et al. categorized commercial white bread as NOVA 4, while other breads were NOVA 3 [90].
  - Kityo et al. categorized most loaf bread (‘*sikppang*’) as NOVA 4 [84].
  - Lane et al. called some breads NOVA 3 (e.g., focaccia, ciabatta, baguette, corn bread) while others were NOVA 4 (e.g., bagels, breadcrumbs, all light breads with added fiber, vitamins, and minerals) [85].
  - Park et al. categorized all bread as NOVA 4 [91].
  - Wolfson et al. called some breads, excluding restaurant breads, NOVA 3 (e.g., sourdough, Italian, naan) [80].
  - Zancheta Ricardo et al. counted traditional Chilean bread as NOVA 3 and industrially produced and packaged bread as NOVA 4 [92].
- Tomato Sauce:
  - Cordova et al. categorized cooked tomato (as an Italian pizza ingredient) as NOVA 1 if it was fresh, but NOVA 4 if on a commercial pizza [76].
  - Pant et al. counted tomato sauce and tomato paste as NOVA 4 [86].
  - Samuthpongtorn et al. called tomato sauce without sufficient detail non-ultra-processed in their main analysis [87].

Depending on how food items were classified, this could cause over- or underestimation of UPF consumption for study participants, for example, where Bonaccio et al. [81] classified all breads as NOVA 3 and Park et al. [91] classified all breads as NOVA 4, regardless of other details. Most situations of difficult classification, such as those described in Table 3, were the result of a lack of information (e.g., lack of known food origin, ingredients, preparation). The information collected in the observational studies was typically less granular than the RCTs, resulting in more possible ambiguity. Furthermore, larger cohort studies may overlap between different countries or continents where food regulations, brand names, and typical food ingredients differ. It is important to recognize that food preparation and ingredient assumptions should be considered within each different region and culture. The EPIC cohort, for example, includes participants from ten Western European countries. Therefore, when classifying data from these participants, different rules were applied based on where the data originated. For example, most breads in France and Italy were considered artisanal and therefore not UPF, whereas breads in the UK were classified as UPFs [93]. The EPIC cohort study, which started collecting data in the 1990s, has the additional challenge of classifying foods with the consideration that formulations have changed over time. To confront this, researchers have used three different scenarios to classify foods according to the NOVA system. The main analysis, which used the “middle-bound” scenario, considered the most likely level of processing for each food. However, foods may be more or less processed so they also calculated “upper-bound” and “lower-bound” scenarios, respectively [76,78].

A guide was published to describe best practices for applying the NOVA classification system, along with an approach to conducting a careful sensitivity analysis to estimate the potential impact on study outcomes from any uncertainty in the classification decisions for each food item [94]. Several studies have recognized the challenges associated with accurately classifying foods and utilized sensitivity analyses to account for possible bias. Hang et al. assigned ambiguous items (i.e., popcorn, soy milk, pancakes or waffles, pie, beef, pork or lamb sandwiches, and tomato sauce) as non-UPFs in the main analysis and as UPFs in a separate sensitivity analysis. This alternative classification, however, did not significantly impact the results [95]. Cho et al. also conducted a sensitivity analysis including foods that were difficult to classify (i.e., pizza/hamburger, chicken, canned tuna, dumpling, yogurt, coffee, and soy milk). The authors reported that the results of the sensitivity analysis were similar to the main results [82]. Cordova and colleagues used data from the EPIC cohort “lower-bound”, “middle-bound”, and “upper-bound” scenarios to repeat analyses and perform a sensitivity analysis. The sensitivity analyses yielded similar results to the main analysis [76]. Although only a small subset of studies conducted a sensitivity analysis, it is important to note that the results of the sensitivity analyses aligned with the main findings. This suggests that results may be comparable between studies even with differences in classification methods, which further supports the body of evidence associating the consumption of UPFs and adverse health effects.

Observational studies cannot determine causal relationships, but large-scale studies can reveal trends in data related to health outcomes. Similar to RCTs, several observational studies included in our analysis found that UPF consumption was associated with obesity and higher BMI [79,87,91], as well as higher sugar intake [89]. The observational studies tracking participants over a long period of time were able to detect associations between UPFs and adverse health outcomes that are not captured in shorter RCT studies. For example, higher intake of UPFs was found to correlate with type 2 diabetes [82], CVD [76,81], and cancer [76,78]. In addition to physical health effects, high UPF consumption was associated with an increased risk of depression and psychological distress [85,87]. Interestingly, high UPF consumption has been associated with inadequate consumption of several micronutrients [83,90], which may partially explain some of the adverse health effects associated with UPFs. 

**Table 3:** Clinical observational studies published within the last year using the NOVA system to categorize UPFs.

References	Food Collection Method and Frequency	Nutritional Program Used	Classification Method	Discrepancy Resolution	Examples of ‘Difficult’ Food Categorization
Ashraf, 2024 [89]	24 h dietary recall using ASA24	ASA24-Canada-2016, Canadian Nutrient File 2015 and FNDDS	Food items were classified according to the NOVA system manually using primarily the “Food Description” variable within the ASA24. The “Food Source” variable (e.g., fast food or vending machine) was also used to identify UPFs. In cases of ambiguity, the least processed category was chosen. Zero kcal foods (e.g., water) not classified and excluded from analysis.	Not described	Cheese was considered NOVA 3, but cheese products categorized as NOVA 4 Mass-produced bacon called NOVA 4
Bonaccio, 2023 [81]	188-item FFQ	Specifically designed software linked to Italian Food Tables	Two researchers independently coded each food into one of four categories. Conservative classification was used for challenging items. Only unequivocal foods were classified as NOVA 4 (e.g., margarine, sweet or savory packaged snacks, etc.). Some uncertain foods were classified using the most common brands in the Italian Food composition Database with the Open Food Facts database.	Discrepancies in classification were discussed with a third researcher and conservative classification was used	Bread was categorized as NOVA 3 Breakfast cereal and biscuits classified using the most consumed brands in the Italian Food composition Database with the Open Food Facts database
Cho, 2024 [82]	103-item FFQ	N/A	Three study researchers classified food items on the FFQ into NOVA categories. The senior author supervised and checked for accuracy. Limited information was available to determine if some items were UPFs, so in this case, they were called non-UPFs and then sensitivity analysis was performed with them as UPFs.	Not described	Items called non-UPFs then UPFs in sensitivity analysis: chicken (e.g., drumstick and wing), canned tuna, dumpling, yogurt, coffee, and soy milk Another sensitivity analysis excluded pizza/hamburgers from the UPF category since they can be made without UPF ingredients
Cordova, 2023 [76] Referred to Huybrechts, 2022 [93]	Country-specific FFQ; combination of FFQ and 7- and 14-day food records were used in Sweden and the UK, respectively	EPIC database	Generic or multi-ingredient foods were decomposed into ingredients. Because data collection started in the 1990s and the food environment has changed over the years, “middle-bound” scenario or the most likely environment was used for food processing.	Not described	Bread in Italy: lower and middle bound assumed NOVA 3—bakery; upper bound assumed NOVA 4—commercial Bread in UK: lower bound assumed NOVA 3—bakery; middle and upper bound assumed NOVA 4—commercial Cooked tomato (as pizza ingredient in Italy): lower and middle bound assumed NOVA 1—fresh; upper bound assumed NOVA 4—commercial pizza
García-Blanco, 2023 [83]	147-item FFQ	N/A	Two researchers independently coded each food into one of four categories based on the NOVA system.	Discrepancies resolved by consensus	Foods that were unknown if they are homemade or industrialized (e.g., pizza, popcorn, lasagna) were classified as UPFs because most traditional foods have been replaced by industrial food products in supermarkets
Houshialsadat, 2023 [90] Referred to Machado, 2019 [96]	Two 24 h dietary recalls, second recall was ≥8 days after the first	Australian Food Composition Database	Two expert evaluators classified foods into one of four categories based on the NOVA system, then a second set of two experts checked classifications. Decisions were made based on lists of ingredients from food packages or company websites. Homemade recipes were disaggregated and classified by underlying ingredients.	Discrepancies were discussed until consensus reached among all researchers	When classification not clear (e.g., cake or cupcake, honey, commercial or homemade), the conservative alternative was chosen (e.g., homemade and disaggregated) In Australia, many commercially produced breads are processed rather than ultra-processed, so coded two commercial white breads as NOVA 4 and the rest as NOVA 3
Kityo, 2023 [84] Referred to methods by Khandpur, 2021 [97]	106-item FFQ	N/A	A nutritionist classified each FFQ item using the NOVA system with slight modification developed by Khandpul et al., then a registered dietitian validated each classification. Mixed dishes or aggregated foods were disaggregated and weights were applied using Korean food recipe information.	When a consensus was not reached, the nutritionist visited stores and websites to verify food labeling information and manufacturing processes and/or referred to previous publications	Most loaf bread (‘*sikppang*’), toast bread, and buns consumed in Korea are mass-produced, packaged, contain additives, and are commonly sold in convenience stores/marts, so categorized as NOVA 4 The major brand of yogurt consumed in Korea is ‘Yoplait’, which is sweetened, flavored, colored, and has artificial additives according to the labeling information, so categorized as NOVA 4 Dumplings, black bean and spicy seafood noodles were disaggregated into basic ingredients and called NOVA 1 or 3
Kong, 2024 [77]	Two 24 h dietary recalls	FNDDS and NNDSR	NHANES food codes were obtained which categorized foods according to NOVA. Homemade dishes with unknown ingredients were classified according to their expected components. Foods lacking sufficient information to determine the degree of processing was usually solved by selecting a lower degree of processing.	Not described	“Yogurt, NFS” was classified as NOVA 1 “Restaurant, Chinese, Sesame Chicken” was coded as “Orange chicken” and classified as “meat” and NOVA 1
Lane, 2023 [85] Referred to methods by Machado, 2019 [96]	121-item FFQ	Nutrient Data Table for Use in Australia 1995	Two authors with Australian food and dietary intake knowledge classified all FFQ food items into NOVA categories. For items that could not be discriminated (e.g., ‘bread’, ‘pasta or noodles’, ‘low fat cheese’, ‘yoghurt’, ‘fruit juice’), the authors referred to the National Nutrition Survey 1995-96 and NNPAS 2011-12 for comparison and decision making. When lacking details, foods were disaggregated and the conservative alternative was chosen (i.e., homemade or processed vs. UPF).	Not described	When classification not clear (e.g., cake or cupcake, honey, commercial or homemade), the conservative alternative was chosen (e.g., homemade and disaggregated) NOVA 3 breads: focaccia, ciabatta, baguette, pane di casa, sour dough, flats (naan, paratha, chapatti, roti, injera, and pita), pumpkin bread, corn bread and tortillas NOVA 4 breads: bagel, breadcrumbs, hot dog breads, fast-food breads, pizza bases, all light breads and with addition of fiber, vitamins, and minerals
Morales-Bernstein, 2024 [78]	Country-specific FFQ; combination of FFQ and 7- and 14-day food records were used in Sweden and the UK, respectively	N/A	Food items were categorized using the NOVA system. Food preparations using traditional methods (e.g., homemade) were disaggregated using standardized recipes.	Not described	Preserved vegetables, legumes and fruits categorized as NOVA 3 Potato products, vegetable spreads and fizzy drinks were categorized as NOVA 4
Pant, 2023 [86] Referred to Machado, 2019 [96] and Lane, 2023 [85]	101-item FFQ	N/A	Food items from the FFQ were classified into one of the four NOVA groups and cross-checked between two independent reviewers. If classification was unclear, the NNPAS 2011-12 was consulted or lesser degree of processing was selected.	Discrepancies were resolved by group consensus	Pizza and peanut butter were classified as NOVA 1 Tomato sauce and tomato paste were classified as NOVA 4
Park, 2024 [91]	One 24 h dietary recall	Standard Food Composition Table by the National Institute of Agricultural Sciences	Two researchers classified each food item using the NOVA system. Product names, manufacturer, and nutritional information used to classify food as accurately as possible.	Items with discrepancies were discussed and resolved by consensus	Most or all fruit jams and canned fruits categorized as NOVA 3 Most or all bread and bakery products categorized as NOVA 4
Price, 2024 [79]	Two 24 h dietary recalls	NHANES Nova 2015–18 database Food coded for NHANES using FNDDS and NNDSR	Food classifications made using underlying ingredients. Foods were categorized using NOVA as UPFs in three ways: (1) using original NOVA methods, (2) excluding ≥25% whole grains from UPFs, and (3) excluding ≥50% whole grains from UPFs.	Not described	Commercial whole-grain bread and ready-to-eat cereals categorized as NOVA 4 reanalyzed as non-UPFs
Samuthpongtorn, 2023 [87] Referred to Hang, 2023 [95]	One FFQ every 4 years between 2003 and 2017	N/A	Three researchers independently assigned each food item to a NOVA group. Foods lacking consensus were discussed with an expert group and additional resources (research dieticians, cohort-specific documents, and online grocery store scans) were used.	Items lacking consensus were discussed with an expert group and additional resources used	Foods lacking sufficient detail (i.e., “popcorn”; “soy milk”; “pancakes or waffles”; “pie, home-baked or ready-made”; “beef, pork, lamb sandwich”; “tomato sauce”) were assigned to a non-UPF group, then later to a UPF group for sensitivity analysis
Sullivan, 2023 [88]	124-item FFQ completed at baseline, year 2, and year 4	Diet History Questionnaire nutrient and food group database; Diet*Cal Analysis Program (version 1.4.3, NCI Epidemiology and Genomics Research Program)	Two researchers independently categorized all items using the NOVA system. Discordantly assigned items were placed in the less-processed group. Sensitivity analysis performed with items assigned to more-processed group.	Not described	Tofu and honey were grouped into UPF categories because they could not be disaggregated from mixed foods
Wolfson, 2024 [80] Refers to Martinez Steele, 2016 [98] and 2023 [99]	Two 24 h dietary recalls, 3‒10 days apart on different days of the week	Food coded using FNDDS and NNDSR	Food items were classified according to the NOVA system using a unique 8-digit food code. Foods likely to be homemade or artisanal were linked to scratch ingredients while foods likely purchased ready-to-eat were not disaggregated.	Not described	Several uncertain breads, such as sourdough, Italian, and naan, excluding from fast-food restaurants, categorized as NOVA 3 Some uncertain breakfast cereals such as corn flakes, frosted corn flakes, puffed rice, and raisin bran categorized as NOVA 3 Some uncertain salty snacks such as chips, crackers, and popcorn categorized as NOVA 3
Zancheta Ricardo, 2023 [92]	24 h dietary recall	SER-24 (CIAPEC)	Three different methods used to identify UPFs based on the NOVA system: (1) using the usual NOVA categories, (2) if they contained at least one ingredient not commonly used in home cooking, and/or (3) cosmetic additives. Food was classified by one dietitian and reviewed by a second dietitian. A third person classified a small random subset of records to verify. Homemade recipes were disaggregated into their components and classified.	Disagreements were discussed and resolved by consensus	Unbranded traditional Chilean bread assigned NOVA 3, while industrially produced, packaged, and branded bread assigned to NOVA 4

Abbreviations: ASA24 = National Cancer Institute’s web-based Automated Self-Administered 24-h Dietary Assessment Tool; UPF = ultra-processed food; FFQ = food frequency questionnaire; NHANES = National Health and Nutrition Examination Survey; EPIC = European Prospective Investigation into Cancer and Nutrition; UK = United Kingdom; NNPAS = Australian National Nutrition and Physical Activity Survey; FNDDS = Food and Nutrient Database for Dietary Studies; NNDSR = National Nutrient Database for Standard Reference; NCI = National Cancer Institute; CIAPEC = Center for Research in Food Environments and Prevention of Nutrition-Associated Diseases; N/A = not applicable.

## 5. Future Directions

### 5.1. Future Directions for Food Classification

The need for further research on UPFs is well recognized, as illustrated by a recent interdisciplinary workshop to define gaps in knowledge and to propose key research questions for future study [61]. Recently, several groups have tackled the challenge of standardization. For example, to enhance the ability to compare results across studies and to increase the quantitative nature of the data, online surveys and databases have been developed, such as a database to estimate food additive intake based on The Food Agricultural Organization/World Health Organization (FAO/WHO) International Food Standards *Codex Alimentarius CXS 192e International Food Standards* [100]. Zancheta Ricardo et al. used 24 h dietary recall data to compare three different methods: (1) the classic method of NOVA based on the extent and purpose of processing, (2) an ingredient method linking lists of ingredients from packaged foods to computer software, and (3) a food additive method as defined by Codex Alimentarius. They found that the foods labeled as UPF varied from 65% (classic method) to 73% (food additive method), and that considering the specific list of ingredients increased the proportion of food products counted as UPF [92]. Canella et al. proposed an alternative approach to defining UPFs as those with cosmetic additives (e.g., flavor enhancers, colors, emulsifiers) and/or critical nutrients (i.e., sugars, sodium, total fat, and saturated fat) in excess on food labels. The agreement between this approach and the NOVA classification was quite high, as 98.8% had a cosmetic additive and/or a nutrient in excess. This promising approach may help to reduce ambiguity in the classification of UPFs and make cross-study comparisons easier [101]. Additional studies are needed that use these techniques along with a measurement of effects on health.

Researchers are also starting to develop web-based tools for food processing classification which should help with study comparability. In 2023, Neri et al. introduced a web-based self-completed 24 h recall tool (called Nova24h) designed to assess dietary intake according to the NOVA food classification system. One-hundred and eighty-six participants of the NutriNet Brasil cohort study completed the Nova24h assessment, which was found to be comparable to the standard 24 h dietary recall method. This is an excellent start to obtaining reproducible categorization, but further studies with more participants, more food items, and in additional countries are needed to determine the full utility of this tool [102]. Martinez Steele et al. presented a reference approach method for the NOVA classification system using 24 h dietary recalls from the 2001–2018 cycles of What We Eat in America (WWEIA), NHANES data, and conducted four sensitivity analyses comparing alternative approaches, to improve standardization across studies. Using the reference approach, they found that UPFs accounted for 58% of dietary energy contribution, while sensitivity analysis showed this ranged from 53% to 60% across approaches [99].

The NOVA classification system has been criticized by some for the uncertainty in the definitions of the four categories and it has been suggested that more traditional nutrient profiling may be more useful [103]. Consumer-friendly web-based nutrition tools have been designed to help individuals make healthier food choices. The Nutri-Score, a front-of-package nutrition labeling system implemented in France and several other European countries, is a user-friendly way to inform consumers of healthy food choices [104]. The NOVA classification system and Nutri-Score have been compared and, although they both provide valuable information, the specific categories do not completely align [105].

### 5.2. Future Directions for Determining UPFs’ Impact on Gut Microbiome and Other Health Outcomes

The sample of RCT and observational studies included in our analysis show a trend of UPFs associated with a number of non-communicable diseases such as obesity, cancer, and CVD [65,67,68,69,76,78,81,82,91]. The purpose and composition of UPF ingredients vary greatly; therefore, different types of UPFs are expected to have different effects on the body (i.e., all UPFs are not equivalent). UPFs often contain high salt and sugar content, which are also associated with the Western diet, so it is difficult to distinguish possible effects of processing itself and other negative traits of UPFs or the Western-style diet [1,106]. Another complication is that UPFs do not exclusively contain unhealthy ingredients. In a cross-sectional analysis by Price et al. using data from over 11,000 individuals in the NHANES study, they distinguished between UPFs that contained whole grains and those that did not and found that UPFs high in whole grains may not significantly contribute to cardiometabolic risk factors [79]. The next steps in our understanding of the effects of UPFs on health is to identify the direct causation and underlying mechanisms. This will require more randomized controlled studies in humans and controlled studies in animals using more standardized food classification methods such as those proposed above, along with sensitivity analyses and detailed methods, so that studies can be easily reproduced. Future studies should also include gut microbiome data, metagenomics, and metabolomics data to identify potential biological signatures for the effects of UPFs.

## 6. Conclusions

In this narrative review, we discuss four original publications that have examined the effects of UPFs on the gut microbiome. There are very few clinical studies in humans with a focus specifically on UPFs and the gut microbiome, as distinguished from other potentially overlapping dietary patterns. Two studies each identified associations between UPF consumption and increased *Prevotella* spp. [17,19] and decreased *Lachnospira* spp. [16,19] and *Ruminococcus* spp. [18,19]. While more studies are needed to draw definitive results on the impact on the gut microbiome, we can also consider the well-known effects of a Western-style diet on the gut microbiome, as the Western diet contains high amounts of processed foods and UPFs. One of the largest barriers to UPF study reliability and reproducibility is overcoming the ambiguity surrounding the NOVA food classification system, so studies that provide very detailed descriptions of their food processing categorization are valuable. Of the studies included in our analysis, approximately 63% and 86% of RCT and observational studies, respectively, did not provide the level of detail included in the publications with the most comprehensive methods. How does that impact our consideration of the broader literature regarding potentially harmful effects of UPFs? 

Even with the limitations we have described, it is hard to deny accumulating evidence of some harmful health effects of some UPFs, but there is quite a gap in the understanding of which UPFs matter the most and what amount of NOVA 4 foods, if any, might be included in a diet otherwise composed of mostly NOVA 1–3 foods without harm. This is an important question when considering ultra-processing that improves food stability, especially in areas of limited resources. Careful meta-analyses that weigh the controls of each study when developing broad conclusions from many studies will be essential. To determine causation and to fully understand mechanisms by which UPF ingredients affect biological functions and microbiota, whose pathways intricately intertwine with host cellular pathways, the details of the methods and classification approaches will matter. Animal studies will provide data that cannot be obtained from human studies. On a positive note, even without understanding all of the effects of all ingredients, a switch to more NOVA 1 foods is likely to improve the health of many individuals in real time, if they can be convinced to switch, while science proceeds at a slower pace. To increase reproducibility, we propose utilizing the methods reviewed here, such as considering food additive types and/or the nutrient content of packaged foods. Studies should also share information about foods that they had difficulties classifying and perform sensitivity analyses to reduce bias. Overcoming these food categorization challenges is imperative for the field to move forward in our understanding of the true biological impacts of UPFs on the gut microbiome and health.

## Supplementary Materials

The following supporting information can be downloaded at https://www.mdpi.com/article/10.3390/nu16111738/s1: Table S1: PubMed search strategy for observational studies; Table S2: PubMed search strategy for randomized controlled trials; Table S3: Characteristics of clinical studies examining UPFs and the gut microbiome; Table S4: Characterization of clinical randomized controlled trials using the NOVA system to categorize UPFs; Table S5: Characteristics of clinical observational studies published within the last year using NOVA system to categorize UPFs. Ref. [107] is cited in Supplementary Materials.
