# Supplementary material for: Ultra-Processed Foods: A Narrative Review of the Impact on the Human Gut Microbiome and Variations in Classification Methods
Source: Nutrients. 2024 Jun 1;16(11):1738. doi: 10.3390/nu16111738 (PMC11174918; doi:10.3390/nu16111738)
Supplement: Supplementary file 1 [file nutrients-16-01738-s001.zip › nutrients-3008615-supplementary.pdf]

Table S1. PubMed search strategy for observational studies (154 results):

Search Number	Search Terms/Combinations
#1	((((food[Title/Abstract]) OR (foods[Title/Abstract])) OR (diet[Title/Abstract])) OR (meal[Title/Abstract])) OR (meals[Title/Abstract])
#2	((ultraprocessed[Title]) OR (ultra-processed[Title])) OR (processed[Title])
#3	(nova[Title/Abstract]) OR (NOVA[Title/Abstract])
#4	((classification[Title/Abstract]) OR (classifying[Title/Abstract])) OR (categories[Title/Abstract])
#5	#3 AND #4
#6	#2 OR #5
#7	#1 AND #6
#8	#1 AND #6 AND (english[Filter])
#9	#1 AND #6 AND ((review[Filter] OR systematicreview[Filter]) AND (english[Filter]))
#10	#8 NOT #9
#11	(study[Title/Abstract]) OR (trial[Title/Abstract])
#12	(((observational[Title/Abstract]) OR (cross-section[Title/Abstract])) OR (cross-sectional[Title/Abstract])) OR (cohort[Title/Abstract])
#13	#11 AND #12
#14	#10 AND #13 AND (2023/1/1:2024/12/31[pdat])

Table S2. PubMed search strategy for randomized controlled trials (77 results):

Search Number	Search Terms/Combinations
#1	((((food[Title/Abstract]) OR (foods[Title/Abstract])) OR (diet[Title/Abstract])) OR (meal[Title/Abstract])) OR (meals[Title/Abstract])
#2	((ultraprocessed[Title]) OR (ultra-processed[Title])) OR (processed[Title])
#3	(nova[Title/Abstract]) OR (NOVA[Title/Abstract])
#4	((classification[Title/Abstract]) OR (classifying[Title/Abstract])) OR (categories[Title/Abstract])
#5	#3 AND #4
#6	#2 OR #5
#7	#1 AND #6
#8	#1 AND #6 AND (english[Filter])
#9	#1 AND #6 AND ((review[Filter] OR systematicreview[Filter]) AND (english[Filter]))
#10	#8 NOT #9
#11	#8 NOT #9 AND (randomizedcontrolledtrial[Filter])
#12	#8 NOT #9 AND (clinicaltrial[Filter])
#13	#11 OR #12
#14	trial[Title/Abstract]
#15	((randomized[Title/Abstract]) OR (randomize[Title/Abstract])) OR (randomly[Title/Abstract])
#16	#14 AND #15
#17	#10 AND #16
#18	#13 OR #17 AND (y_10[Filter])

Table S3. Characteristics of clinical studies examining UPFs and the gut microbiome

Reference & Country	Study Design (Cohort)	Study Population, Sample Size (n)	Food Collection Method & Frequency	How UPFs Were Compared in the Study	Main Results	Metabolite Changes Associated with UPFs		Clinical or Dietary Quality Outcomes Associated with UPFs	
						Increased ↑	Decreased ↓	Increased ↑	Decreased ↓
**Atzeni, 2022** [17]<br><br>Spain	Cross-sectional analysis of an intervention study (substudy of the PREDIMED-Plus trial)	Older adults aged 55–75 years with overweight/ obesity and metabolic syndrome (645)	FFQ	Proportions of consumed UPFs categorized in tertiles: low UPF (first tertile, n=215), medium UPF (second tertile, n=217), and high UPF (third tertile, n=213) consumption	UPF consumption positively associated with lower MedDiet adherence and higher total energy intake, however few associations between the gut microbiota and UPFs observed.	No significant differences in insulin, cholesterol, TG, or glycated hemoglobin between tertiles. No significant differences between bacterial taxa and cardiovascular risk factors.		No significant differences in body weight, waist cir, or BMI between tertiles.	
**Cuevas-Sierra, 2021** [16]<br><br>Spain	Cross-sectional analysis of an intervention study (Obekit trial)	Caucasian adults (359)	FFQ	"low" UPF consumption group (<3 servings/day; W:57, M:39) vs. "high" UPF consumption group (>5 servings/day; W:66, M:24)	Consumption of UPFs may affect gut microbiota differently in men and women.	**Whole population:** TG (p=0.004)<br><br>**Men:** TG (p=0.006), ALT (p=0.04), and TNF-α (p=0.009)	**Whole population:** HDL-c (p=0.04)<br><br>**Men:** HDL-c (p=0.01)	**Whole population:** BMI (p=0.02), body weight (p=0.002), waist cir (p=0.02), hip cir (p=0.01), and fat mass (p=0.01)<br><br>**Women:** depression (p=0.01), anxiety (p=0.02), weight (p=0.04), and hip cir (p=0.03)<br><br>**Men:** BMI (p=0.02), weight (p=0.03)	**Whole population:** adherence to MedDiet (p<0.001)<br><br>**Women:** adherence to MedDiet (p<0.001)<br><br>**Men:** adherence to MedDiet (p=0.006)
**Fernandes, 2023** [18]<br><br>Brazil	Cross-sectional	Women (59)	Three 24-hr dietary recalls	NOVA group 1 (unprocessed or minimally processed) vs. group 4 (UPFs)	Consumption of UPFs associated with leptin resistance and changes in the gut microbiota composition.	leptin/fat mass (p=0.049)		No significant associations between UPF and other anthropometric or clinical variables.	

**García-Vega, 2020** [19] Colombia	Cross-sectional cohort study	Community-dwelling Colombians (441)	One (347/441) or two (94/441) 24-hr dietary recalls	Ultra-processed vs. not ultra-processed	Diet quality and intake of food groups and nutrients showed associations with the gut microbiota. Diets enriched with plant-derived foods trended towards having a more diverse gut microbiota and increased levels of SCFA-producing bacteria. Higher consumption of UPFs associated with more inflammatory gut bacteria.	No metabolites measured.	Diet quality indices (HEI and GABA) higher in older participants (aged 41-62 years vs. 18-40 years), while UPF intake is deceased.

Abbreviations: UPF = ultra-processed food; FFQ = food frequency questionnaire; BMI = body mass index; W = women; M = men; ALT = alanine aminotransferase; TNF-α = tumor necrosis factor alpha; HDL-c = high density lipoprotein cholesterol; MedDiet = Mediterranean diet; SCFA = short-chain fatty acid; HEI = healthy eating index; GABA = Colombian Food-Based Dietary Guidelines index; cir = circumference; TG = triglycerides

Table S4. Characterization of clinical randomized controlled trials using the NOVA system to categorize UPFs

Reference	Country	Study Design (Cohort)	Study Population, Sample Size (n)	Intervention Group	Control Group	Duration	Food provided?	Main Results
**Capra 2024 [31]**	USA	Parallel, outpatient controlled feeding trial	Adults aged 40–65 years with stable weight and BMI ≤35 kg/m² (Anticipated n=17/group)	UPF (81%) diet	Non-UPF (0%) diet	8 weeks (2 week lead-in diet (59% UPF diet) then 6-week study diet)	Yes	Diet palatability ratings similar between UPF and non-UPF diet. Lower cost for UPF vs. non-UPF diet ($20.97/day vs. $40.23/day). Additive exposure analysis revealed soy lecithin, citric acid, sorbic acid, and sodium citrate among the most commonly consumed additives.
**Fagherazzi, 2021 [65]**	Brazil	Secondary analysis of a parallel study (DASDIA)	Pregnant women with pre-gestational diabetes mellitus (49)	DASH diet, adapted to Brazilian culture	Standard dietary recommendations for patients with diabetes mellitus	Minimum 8 weeks	Some foods depending on diet group	Women following the DASH diet exhibited improved glycemic control (p=0.01) and consumed significantly fewer UPFs compared to standard diet (p=0.01).
**Fangupo, 2021 [67]**<br><br>**Refers to Taylor, 2011 [107]**	New Zealand	Secondary analysis of 4-arm parallel study (Prevention of Overweight in Infancy)	Mothers, age ≥16 years, and their children born May 2009–December 2010 (669)	SWCC+ Food/Activity, SWCC+ Sleep, SWCC+ Food/Activity+ Sleep	Standard Well Child Care (SWCC)	60 months	No	Average energy intake from UPF at 12, 24, and 60 months old was 45%, 42%, and 51%, respectively. Maternal obesity associated with higher UPF intake at 12 months.
**Gonzalez-Palacios, 2023 [68]**	Spain	Prospective, longitudinal analysis (PREDIMED-Plus)	Adults aged 55-75 with overweight/ obesity and metabolic syndrome (5,373)	Energy-restricted MedDiet + EVOO, PA promotion, and behavioral support	Unrestricted energy MedDiet + EVOO	12 months	Some, both groups received EVOO and almonds	Volunteers significantly reduced UPF consumption after 6 and 12 months compared to baseline (104.1 ± 116.9 g/day and 100.7 ± 114.6 g/day vs. 159.4 ± 155.1 g/day). Highest UPF quartile associated with several cardiometabolic risk factors, including weight, BMI, waist circumference and fasting glucose, compared to the lowest UPF quartile.
**Hall, 2019 [62]**	USA	Cross-over controlled-feeding trial	Healthy weight stable adults (20 inpatient)	UPF diet (80% UPF)	Unprocessed food diet (0% UPF)	4 weeks (2 weeks per diet)	Yes	UPF diet associated with elevated consumption of energy, carbohydrates, and fat, without a corresponding increase in protein intake. Changes in weight positively correlated with energy intake (+0.9 ± 0.3 kg, p=0.009 during UPF diet vs. -0.9 ± 0.3 kg, p=0.007 during unprocessed diet).
**Konieczna, 2021 [69]**	Spain	Prospective, longitudinal analysis of a parallel clinical trial (PREDIMED-Plus)	Spanish adults aged 55–75 with overweight/obesity and metabolic syndrome (1,485)	Energy-restricted MedDiet, PA promotion, and behavioral support	Non-intensive recommendations on unrestricted energy MedDiet and traditional healthcare	12 months	Some, both groups received EVOO and almonds	After adjusting for multiple variables, modeling revealed that a 10% increase in daily consumption of UPF was significantly links to higher levels of visceral fat (p<0.0001), an increased android-to-gynoid fat ratio (p=0.031), and elevated total fat content (p<0.0001) over a period of 12 months.
**O'Connor, 2023 [63]**<br><br>**Refers to Hall, 2019 [62]**	USA	Secondary analysis of a cross-over controlled-feeding trial	Healthy weight stable adults (20 inpatient)	UPF diet (80% UPF)	Unprocessed food diet (0% UPF)	4 weeks (2 weeks per diet)	Yes	Evaluation of metabolites in plasma and urine showed 257 out of 993 plasma and 606 out of 1279 24-hr urine metabolites differed between the UPF and unprocessed diets. Twenty-one known and nine unknown metabolites differed between diets across all timepoints and biospecimen types (plasma, 24-hr urine, spot urine).

**Phillips, 2021 [70]**	Switzer-land	(A) Observation phase; (B) randomized parallel phase (SwissChrono-Food trial)	(A) Adults (213); (B) Adults with eating duration >14 hrs and at least one metabolic syndrome component (54)	(B) 12-hr time-restricted eating (TRE)	(B) Standard dietary advice (SDA)	(A) 4 weeks; (B) 6 months	No	After 6 months, TRE group lost 1.6% of initial body weight compared to no weight loss in the SDA group, but difference was not significant. Mobile apps show promise in tracking eating behaviors and better understanding the relationship between eating habits and metabolic health.
**Rego, 2023 [64]**	USA	Cross-over controlled-feeding trial	Weight stable adolescents and young adults aged 18-25 years (32, estimated)	High UPF (81% kcals from UPF) diet	Non-UPF (0% kcals from UPF) diet	8 weeks (2 weeks each diet with 4 week washout in-between diets)	Yes	Trial currently recruiting/underway. Primary aim is to determine influence of UPF consumption on brain reward response, while a secondary aim is to examine influence of UPF consumption on ad libitum energy intake and food selection.
**Sneed, 2023 [66]**	USA	Secondary analysis of a parallel study (Growing Right Onto Wellness)	Children, aged 3-5 years, with normal weight to overweight, but not obese, and who qualified for at least one service for underserved populations (610 parent-child pairs)	GROW Healthier Group: (1) group sessions that meet once weekly for 3 months, phone call coaching for 9 months, and monthly cues to action for healthy activities for 24 months, and (2) group sessions that meet six times over 3 years	GROW Smarter Group: group sessions that meet six times over 3 years	3 years	No	When comparing NOVA categorization between coding pairs, 84.5% (2,619 items) of foods had concordant categorization, 11.2% (347 items) had discordant categorization, and 4.3% (134 items) were "I don't know" from one or both coders. Fruits, condiments/spices, ready-to-eat foods, and grains had highest rates of discordance. UPFs had higher added-sugar-to-calorie ratios and lower protein-to-calorie ratios than minimally processed foods. UPFs made up 62% of daily calories and negatively correlated with HEI score.

Abbreviations: MedDiet = Mediterranean diet; EVOO = extra-virgin olive oil; UPF = ultra-processed food; FFQ = food frequency questionnaire; HEI = healthy eating index; NDS-R = Nutrition Data System for Research; DASH = Dietary Approaches to Stop Hypertension; HIV = human immunodeficency virus, PA = physical activity; DASDIA = study aimed at testing effect of the DASH diet on perinatal outcomes in pregnant women with pre-gestational diabetes mellitus

Table S5. Characteristics of clinical observational studies published within the last year using NOVA system to categorize UPFs

Reference	Country	Study Design (Cohort)	Study Population, Sample Size (n)	Duration	Main Results
**Ashraf, 2024 [89]**	Canada	Cross-sectional (Guelph Family Health Study)	Families with children aged 1.5–5 years residing in Guelph-Wellington in Ontario, Canada (365 parents; 267 children)	N/A	Greater dietary contribution of UPFs associated with increased intake of total and free sugars in a cohort of Canadian families with preschool-aged children.
**Bonaccio, 2023 [81]**	Italy	Prospective (Moli-sani Study)	Adults living in Molise, aged 35 years or older, with type 2 diabetes (1,065)	Median follow-up 11.6 years	Higher consumption of UPFs in participants with type 2 diabetes associated with reduced survival and higher cardiovascular disease mortality.
**Cho, 2024 [82]**	Korea	Population-based prospective cohort (Korean Genome and Epidemiology Study Ansan–Ansung cohort)	Adults aged 40–69 years without type 2 diabetes or diabetic medication (7,438)	Median follow-up 15 years	High intake of UPFs, particularly ham/sausage, instant noodles, ice cream, and carbonated beverages, associated with increased risk of type 2 diabetes, while intake of candy/chocolate was associated with decreased risk in Korean adults.
**Cordova, 2023 [76]**	7 European Countries (Denmark, Italy, UK, Germany, Spain, Sweden, Netherlands)	Prospective cohort study (sub-study of the EPIC cohort)	Adults aged 35–74 years, free of cancer, cardiovascular disease, and type 2 diabetes at recruitment (266,666)	Median follow-up 11.2 years	Higher consumption of UPFs associated with a higher risk of multimorbidity for cancer and cardiometabolic diseases.
**García-Blanco, 2023 [83]**	Spain	Prospective, cross-sectional (SENDO project)	Children aged 4–5 years that reside in Spain (806)	N/A	High consumption of UPFs in children associated with increased odds of inadequate intake of micronutrients.
**Houshialsadat, 2023 [90]**	Australia	Cross-sectional (National Nutrition and Physical Activity Survey)	Australians aged ≥2 years (12,153)	N/A	UPFs contribution to energy quintiles negatively associated with dietary diversity and micronutrient intake.
**Kityo, 2023 [84]**	Korea	Population-based prospective cohort (Korean Genome and Epidemiology Study-Health Examinees cohort)	Adults aged 40–69 years at baseline using sites participating in the study for more than 2 years (113,576)	Median follow-up 10.6 years	UPF intake not associated with all-cause, cancer or CVD mortality. Ultra-processed red meat and fish intake in both sexes, and milk and soymilk drink intake for men positively associated with all-cause mortality.
**Kong, 2024 [77]**	USA	Cross-sectional (using data from NHANES 2011-2018)	Adults aged 20–59 years (10,255)	N/A	Increased UPF consumption negatively impacted muscle mass in adults and represents a potentially significant driving factor for sarcopenia.
**Lane, 2023 [85]**	Australia	Prospective cohort (Melbourne Collaborative Cohort Study)	Adults aged 27–76 not taking medication for anxiety or depression at baseline (23,299)	15 year follow-up	Highest intake of UPF at baseline associated with elevated psychological distress, measured by the ten-item Kessler Psychological Distress Scale as an indicator of depression, compared to individual with the lowest UPF intake.

**Morales-Bernstein, 2024 [78]**	9 European Countries (Denmark, France, Germany, Italy, Norway, Spain, Sweden, UK, Netherlands)	Prospective cohort (sub-study of the EPIC cohort)	Individuals free of cancer at recruitment (450,111)	Mean follow-up 14.13 ± 3.98 years	A 10% gram/day higher UPF consumption associated with greater risk of head and neck cancer and oesophageal adenocarcinoma. Only a small proportion (up to 15%) of this effect mediated by adiposity measured by BMI and waist-to-hip ratio.
**Pant, 2023 [86]**	Australia	Prospective cohort (Australian Longitudinal Study on Women's Health)	Women aged 50–55 and free of CVD at baseline (10,006)	15 year follow-up	In women, higher UPF intake (mean 42.0% vs. 14.2% total dietary intake) associated with increased hypertension, but not occurrence of CVD or all-cause mortality.
**Park, 2024 [91]**	Korea	Cross-sectional (using data from KNHANES 2016–2020)	Adults aged ≥19 years and free of cancer (22,688)	N/A	Higher total daily UPF intake associated with an increased risk for metabolic syndrome, hypertension, and abdominal obesity.
**Price, 2024 [79]**	USA	Cross-sectional (using data from NHANES 2015–2018)	Adults aged ≥20 years (11,288)	N/A	High UPF intake positively associated with increased weight, waist circumference, and weight-to-height ratio. After removing UPFs with ≥25% and ≥50% whole grain, UPF intake correlated with increased C-reactive protein but reduced cholesterol.
**Samuthpongtorn, 2023 [87]**	USA	Prospective cohort (Nurses' Health Study II 2003–2017)	Women aged 42–62 years and free of depression at baseline (31,712)	14 years	High UPF intake associated with increased BMI, smoking, prevalence of comorbidities, and risk of depression (esp. when consuming artificial sweeteners and artificially sweetened beverages).
**Sullivan, 2023 [88]**	USA	Prospective cohort (Chronic Renal Insufficiency Cohort)	Adults aged 21–74 with reduced estimated glomerular filtration rate (i.e., eGFR 20-70 mL/min/1.73 m^2) (2,778)	Median follow-up 7 or 14 years	Greater UPF intake associated with trend for chronic kidney disease progression, esp. in earlier stages, and higher risk of all-cause mortality.
**Wolfson, 2024 [80]**	USA	Cross-sectional (using data from NHANES 2007-2010)	Adults aged ≥20 years (9,491)	N/A	Frequency and time spent cooking at home associated with lower UPF intake and higher intake of unprocessed or minimally processed foods.
**Zancheta Ricardo, 2023, [92]**	Chile	Cross-sectional secondary analysis (Food Environment Chilean Cohort)	Preschoolers aged 3–6 years in low-to-middle income households with dietary data (958)	N/A	Searching for all possible markers of UPF in the list of ingredients increased the proportion of identified UPFs from 65% to 73%.

Abbreviations: UPF = ultra-processed food; NHANES = National Health and Nutrition Examination Survey; EPIC = European Prospective Investigation into Cancer and Nutrition; MINNADE = Ministry of Health, Labor and Welfare-sponsored Nationwide Study on Dietary Intake Evaluation; CVD = cardiovascular disease; SENDO = Seguimiento del Nino para un Desarrollo Optimo/Follow-up of Children for Optimal Development; UK = United Kingdom; KNHANES = Korea National Health and Nutrition Examination Survey
